# Manual of Bacteriology

**Published:** 1887-12

**Authors:** 


*Manual of Bacteriology.* By EDGAR M. CROOKSHANK, M.B. (Lond.) Pp. 439. London: H. K. Lewis. 1887.

*Photography of Bacteria.* By EDGAR M. CROOKSHANK, M.B. (Lond.) Pp. 64. With 86 Photographs reproduced in Autotype. London: H. K. Lewis. 1887.

These two sumptuous volumes may be looked upon as the best exposition of Bacteriology at present before the English reading public. Medical men in particular owe Dr. Crookshank a debt of gratitude, not only for the clear and admirable treatment of the subject which he has presented to them, but also for the elaborate and necessarily expensive illustrations. As regards *Photography of Bacteria*, we doubt whether the very moderate price charged for the book will cover the primary expense in producing it; the work must be, in some degree at least, looked upon as a free gift to the profession.

The *Manual of Bacteriology* is essentially a second edition of the *Introduction to Practical Bacteriology*, already favourably noticed in these pages. The few months left to the author for the production of a second edition have been most usefully employed in revising and bringing up to date the first edition; in rearranging the species and recasting the systematic portions; and, last but not least, in writing additional chapters on the "General Morphology and Physiology of Bacteria, upon Antiseptics, and Disinfectants and Immunity." Seventy-three new illustrations have been added. A very full and well-arranged Bibliography has been provided.

The *Photography of Bacteria* may be taken as an illustrated supplement to the *Manual*, while the accompanying letterpress is a clear and well-written account of the most modern methods employed in photo-micrography. The author has no need to apologise for these plates; they are certainly the best which we have seen. Many of them are as clear, and give as definite outlines, as hand-drawings; while of the others, their unmistakable fidelity to nature, their beautiful printing, and admirable selection from what are evidently model specimens, are of such value that we should not willingly exchange them for any hand-drawn illustrations which we have seen.

We most heartily congratulate both author and publisher on the production of these books; and we look forward with pleasant anticipation to much good work which will issue from the Bacteriological Laboratory at King's College, which has been partly created, and is ably presided over, by the talented author of these works.

